# Characterising Functional Venom Profiles of Anthozoans and Medusozoans within Their Ecological Context

**DOI:** 10.3390/md18040202

**Published:** 2020-04-09

**Authors:** Lauren M. Ashwood, Raymond S. Norton, Eivind A. B. Undheim, David A. Hurwood, Peter J. Prentis

**Affiliations:** 1 School of Biology and Environmental Science, Science and Engineering Faculty, Queensland University of Technology, Brisbane, QLD 4000, Australia; 2 Medicinal Chemistry, Monash Institute of Pharmaceutical Sciences, Monash University, 381 Royal Parade, Parkville, Victoria 3052, Australia; 3 ARC Centre for Fragment-Based Design, Monash University, Parkville, Victoria 3052, Australia; 4 Centre for Biodiversity Dynamics, Department of Biology, Norwegian University of Science and Technology, 7491 Trondheim, Norway; 5 Centre for Ecological and Evolutionary Synthesis, Department of Biosciences, University of Oslo, PO Box 1066 Blindern, 0316 Oslo, Norway; 6 Centre for Advanced Imaging, University of Queensland, St Lucia, QLD 4072, Australia; 7 Institute of Future Environments, Queensland University of Technology, Brisbane, QLD 4000, Australia

**Keywords:** Cnidaria, Anthozoa, Medusozoa, venom, toxins, transcriptomics, proteomics, spatiotemporal expression, ecology

## Abstract

This review examines the current state of knowledge regarding toxins from anthozoans (sea anemones, coral, zoanthids, corallimorphs, sea pens and tube anemones). We provide an overview of venom from phylum Cnidaria and review the diversity of venom composition between the two major clades (Medusozoa and Anthozoa). We highlight that the functional and ecological context of venom has implications for the temporal and spatial expression of protein and peptide toxins within class Anthozoa. Understanding the nuances in the regulation of venom arsenals has been made possible by recent advances in analytical technologies that allow characterisation of the spatial distributions of toxins. Furthermore, anthozoans are unique in that ecological roles can be assigned using tissue expression data, thereby circumventing some of the challenges related to pharmacological screening.

## 1. Introduction to Animal Venoms

Within the animal kingdom, biotic interactions such as predation and competition are key driving forces for the evolution of species [1,2]. Traits that enhance the success of these interactions, and thus increase survival, are diverse and widespread. The utilisation of venom during interactions with prey, predators and competitors is one such trait, and has evolved on over a hundred separate occasions across at least eight animal phyla [3]. Venomous animals have received considerable interest throughout history, with Aristotle’s “Historia Animalium” serving as the oldest surviving document that references venomous creatures [4,5]. While initial investigations of animal venoms were prompted by the need to develop antivenom strategies [6], studying these complex chemical cocktails has resulted more recently in toxins being employed as molecular tools and as potential candidates for pharmacological development [7,8,9]. This renewed interested in toxins has also enhanced understanding of venom regulation and production.

By definition, venom is a collection of molecules that, when introduced into another animal via a wound, antagonistically interferes with its physiological processes [10]. Venoms typically contain an assortment of salts, amino acids, neurotransmitters, and bioactive proteins and peptides, collectively referred to as toxins [10,11,12,13,14]. Proteins and peptides typically make up the bulk of toxins, and have evolved from physiological proteins and peptides that have been functionally recruited into venoms. However, the relative importance of the various underlying genetic mechanisms governing the compositional evolution of venoms is still poorly understood [15,16]. Venoms are delivered to their victims through specialised delivery structures, which are extremely diverse across venomous lineages [3]. In the vast majority of venomous lineages, these delivery structures are connected to a single set of venom producing tissue(s), forming what is known as centralised venom systems [3]. Alternatively, cells capable of autonomous venom release are distributed throughout the entire body of some venomous organisms, as observed in phylum Cnidaria [17]. Regardless of their composition or associated structures, animals may employ venom as a chemical weapon in instances of prey capture, defence against predators, intraspecific competition, and a number of other diverse functions [3].

As venom is critical to the livelihood of venomous animals, natural selection has resulted in diverse strategies to regulate toxin production and enhance toxin suitability for specific ecological roles. Most venomous animals employ these chemical weapons primarily in order to immobilise prey and/or facilitate feeding [3]. Conversely, venoms may also serve a defensive role by causing intense, instantaneous and localised pain [11,18,19,20]. The same venom is often used for both types of encounters, with toxins that target neuronal communication capable of eliciting both pain and paralysis [21,22,23]. However, this is not the case in the assassin bug and cone snails, which exhibit regionalisation in venom production, within either the same venom duct or distinct anatomical structures [22,24]. This partitioning allows the production of separate venoms for offensive and defensive interactions, thereby reducing the number of toxins depleted during each encounter [22,24]. The ability to produce multiple venoms is evidently beneficial, although to date this has only been observed in a limited number of organisms with a centralised venom system. In cnidarians, there is no centralised morphological structure responsible for the production of venom, and the mechanisms responsible for producing multiple venoms are likely to be more nuanced [16,25]. Furthermore, given the distribution of venom apparatus across multiple functional anatomical tissues, this phylum provides a unique opportunity to ascertain the ecological roles of venom.

## 2. Phylum Cnidaria

Phylum Cnidaria is the most ancient known venomous lineage, having emerged at least 600 million years ago, and includes over 10,000 species [26]. Remarkably, however, only 273 toxins from this phylum are recorded in the ToxProt database [27] as of mid-February 2020. Cnidarians (sea anemones, corals, jellyfish, myxozoans and hydra) are unique among other venomous phyla in that venom production often occurs throughout the entire organism rather than in a single or limited number of discrete anatomical structures. Nematocysts are the highly specialised organelles secreted by the Golgi apparatus that are responsible for storing and discharging venom [17]. The presence of these single-use venom delivery structures is the distinguishing feature of this phylum [17,28]. 

**Figure 1:**
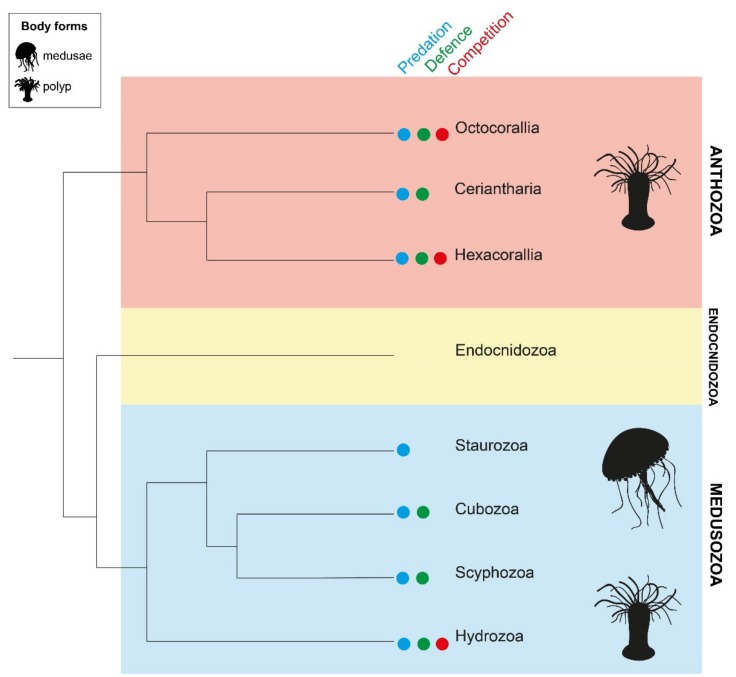
Relationships among cnidarian lineages, Anthozoa (red), Endocnidozoa (yellow) and Medusozoa (blue), based on phylogenomic reconstruction [29]. Sessile polyp forms are observed in both Anthozoa and Medusozoa, while mobile medusae are confined to the Medusozoa (black symbols on right). The presence of venom components in Endocnidozoa and their functions have not yet been verified [30]. In the other classes, it is recognised that venom is utilised as a tool during both predation (blue circle) and defence (green circle). However, venom is only known to be deployed during intraspecific competition (red circle) by Octocorallia, Hexacorallia and Hydrozoa. The ecological significance of venom in some classes requires further characterisation.

Recent phylogenomic analyses have resolved three major cnidarian lineages—Anthozoa, Endocnidozoa and Medusozoa (Figure 1) [29]. Class Anthozoa is a monophyletic clade that is further divided into subclasses Octocorallia (soft corals, sea pens, sea fans), Ceriantharia (tube anemones) and Hexacorallia (zoanthids, sea anemones, true coral, corallimorphs). Scyphozoans (true jellyfish), staurozoans (stalked jellyfish), cubozoans (box jellyfish) and hydrozoans (hydroids, hydromedusae, siphonophores) collectively form clade Medusozoa. Endocnidozoa is comprised exclusively of the freshwater and marine obligate parasites, Myxozoa and Polypodiozoa; this clade possesses a number of cnidarian-restricted genes and nematocyst-homologous structures (polar capsules) but as yet no toxin families have been detected [30]. Traits such as symbiosis, coloniality and life cycle vary within and among cnidarians classes. Solitary and colonial forms are observed across all three clades. Hosting of photosynthetic endosymbionts occurs in a subset of species from Anthozoa and Medusozoa but not Endocnidozoa [29]. A complex life cycle involving both sessile polyp and mobile medusa stages is restricted to Medusozoa but appears to have been lost in class Staurozoa [29]. This high level of diversity within Cnidaria has evolved over at least 600 million years and has implications for the regulation of venom production.

Venom was previously thought to be utilised principally for predation in Cnidaria [11,31]. However, cnidarians are now identified as one of only two phyla known to use venom for all three of the major ecological functions of venom, namely predation, defence and intraspecific competition [3,32]. The extent to which an organism relies upon venom as a defensive strategy is highly variable even within groups [33,34]. In Hexacorallia, for instance, nematocyst discharge in response to simultaneous mechanical and chemical stimuli was observed in all actiniarian species, only 40% of zoanthid species, but no corallimorphians that were tested [33]. These data were substantiated by the field observation that reef fish consistently refused to consume cnidarians with nematocyst defences but not their defenceless counterparts [33]. Likewise, only some hydroid species are dependent upon nematocyst envenomation for protection from predators [34]. Alternate chemical defence via noxious secondary metabolites has also been documented in Anthozoa and Hydrozoa, particularly in species that inhabit coral reefs [33,34]. Within these two classes, the selective utilisation of venom for interspecific or even intraspecific combat has also been observed [35,36,37,38]. Evidence for roles of venom in other classes is limited, with Staurozoa and Ceriantharia remaining the most understudied cnidarian classes to date [39]. Thus, while venom appears to be ubiquitously employed during feeding in anthozoans and medusozoans, further characterisation is required to understand the spectrum of venom usage across the diverse life histories of Cnidaria.

## 3. Venom Evolution across Cnidaria

Venom composition differs substantially among cnidarian classes, with the classes Scyphozoa, Hydrozoa and Anthozoa sharing only six proteins among their soluble nematocyst proteins, which likely have largely house-keeping functions [40]. The proportion of shared protein content is significantly lower for nematocyst proteins (2%) compared to the total proteome (15%) [40]. While venoms of scyphozoans and hydrozoans exert similar biochemical effects, sea anemone venom is unique in that it consists predominantly of peptide neurotoxins [40,41]. However, whether an abundance of neurotoxins is characteristic of anthozoan venom cannot be determined until the taxonomic bias in available data is addressed and knowledge of coral venoms improves. Furthermore, comparison of soluble nematocyst proteomes from eight cnidarian species indicates that approximately one third of all toxin protein families identified are present in both Anthozoa and Medusozoa, although no representative of Staurozoa was included [42]. Of the remaining toxin families, four were taxonomically restricted to a single class and 15 were absent in at least one class, and there was no correlation between toxin family absence and presence and phylogenetic relatedness [42]. In fact, the reported loss of numerous toxin families in Cubozoa was associated with an erroneous phylogenetic reconstruction that placed Cubozoa external to Anthozoa and Medusozoa [42]. The incorrect placement of Cubozoa, however, is likely to be a consequence of using presence/absence variation of only known toxins from the ToxProt database for their phylogenetic reconstruction.

**Figure 2:**
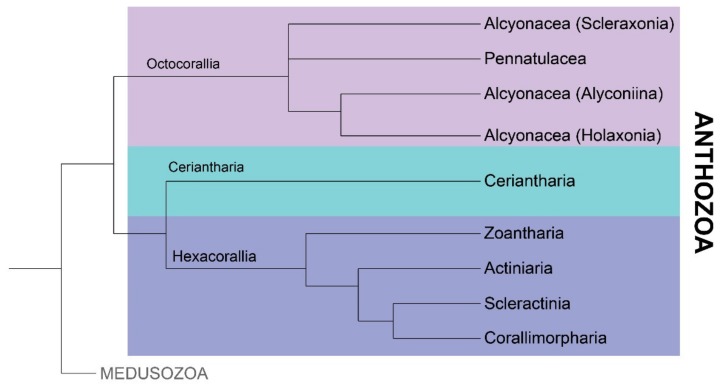
Proposed phylogeny of class Anthozoa based on recent analysis by Kayal et al. [29]. Subclass Octocorallia contains sea pens (order Pennatulacea) and soft corals (order Alcyonacea), while all tube anemones are found in subclass Ceriantharia. Zooanthids (order Zoantharia), sea anemones (order Actiniaria), stony corals (order Scleractinia) and corallimorphians (order Corallimorpharia) belong to subclass Hexacorallia.

Within subclass Hexacorallia, actiniarians (Figure 2) show the greatest biological and anatomical diversity [43]. Sea anemones are distributed across almost every marine environment, from the depths of the oceans to coastal intertidal zones, and from tropical waters to Antarctica [43,44,45]. Their success lies in part in their ability to respond to different environmental pressures [46]. Within actiniarians, ecological interactions and environmental conditions are key drivers of the expression of toxin genes, rather than the retention and expansion of gene families [16,47,48]. Comparative analysis supports the minimal impact of environmental factors on the toxin gene complement, revealing that closely related cnidarian species have more similar toxin gene complements than those that share an ecological niche [16]. Moreover, phylogenetic investigations of sequence variation in cnidarian toxin genes consistently report that toxin gene distribution correlates with species relatedness [49,50,51,52]. These results suggest that speciation is an important driver of toxin gene complement and sequence variation. However, the influence of ecological factors on toxin expression results in dynamic spatial and temporal patterns of venom composition [16,47,48].

## 4. Geographic, Ontogenetic and Prey-Associated Venom Variation

Recent investigations of medically important species such as the box jellyfish, *Chironex fleckeri,* have facilitated exploration of interspecies patterns of venom composition. *C. fleckeri* (class Cubozoa), is regarded as the most lethal jellyfish in the world and is associated with approximately 70 recorded fatalities since 1884 in Australia [53,54]. Comparison of Queensland and Western Australian populations revealed geographic heterogeneity in the composition and potency of their venom arsenal [55]. Similar variability in venom protein content was observed in the scyphozoan giant jellyfish, *Nemopilema nomura*, sourced from a number of locations in the Yellow Sea [56]. In contrast, when clonal fragments of the scleractinian coral, *Tubastraea coccinea,* were reciprocally transplanted between inshore and offshore sites for a six week period, no changes to the abundance and composition of recognised toxins were detected, despite altered expression of non-toxin peptides [57]. Whether this unchanged venom profile is a consequence of similarity in biotic communities between the two locations, the short duration of this study, or is a common attribute of corals and other sessile cnidarians, remains to be determined.

An ontogenetic-driven dietary shift compounds venom complexity in *C. fleckeri*; as medusae mature, they begin preying upon fish in addition to crustaceans. This transition is accompanied by an increase in mastigophores (large-volume nematocysts) and an increased diversity of toxin peptides, which together produce a potent venom specialised for targeting fish [58,59]. Similarly, the number and volume of heteroneme nematocysts, including mastigophores, were found to increase in siphonophore tentilla with a diet of fish compared to those with a diet of copepods [60]. The venom from cubozoan species that consistently prey upon shrimp is also capable of eliciting death in fish, but the amount of venom required exceeds the surface area of both nematocyst-laden tentacles and their prey, making it impractical for them to subdue fish [58]. Hydrozoans also appear to undergo dramatic shifts in venom composition across life stages, and changes in nematocyst type have even been observed in multiple cubozoan species during the transition from polyp to medusa, independent of dietary shifts [61,62,63].

An altered venom protein profile in the absence of nematocyst variation also occurs in the jellyfish *Carukia barnesi* [64,65]. Augmentation of neckchieves, nematocyst bands within tentacles that are postulated to function as a prey attractant, is observed in mature *C. barnesi*, as is an increase in frequency of twitching of this structure to actively lure fish [65,66,67]. These morphological changes provide preliminary evidence that a difference in predatory behaviour accompanies a shift in venom composition during the maturation of some cubozoan species. Thus, a combination of changes to venom and nematocyst profiles, as well as feeding behaviours, which occur during metamorphosis, facilitates a change in diet in cnidarian species.

This intraspecific variability in cnidarian venoms is reminiscent of similar geographical and ontogenetic changes observed in highly-studied terrestrial venomous taxa, particularly snakes, where venom composition is often attributed to changes in diet [68,69,70,71,72,73]. Therefore, it can now be appreciated that within phylum Cnidaria, different patterns of venom composition emerge in response to changes in ecological factors associated with life history transitions, such as diet, and that these patterns are probably driven by changes in gene regulation and expression, as observed in snakes [74].

## 5. Colonial Regionalisation and Functional Divisions

Cnidarians can exist as solitary polyps or as colonies, with coloniality common among corals, zoanthids, hydroids and also select sea anemones [29]. Colonies are comprised of many physically connected individuals, termed zooids, formed through asexual reproduction but with variable morphological forms and functional responsibilities [75,76]. Perhaps the most well-known toxin associated with colonial cnidarians is palytoxin. First isolated from the *Palythoa* genus around 1970, and since isolated in *Heteractis crispa*, this non-proteinaceous toxin poses a serious human health threat, with exposure having the potential to rapidly cause death via heart failure [77,78,79]. In marine environments, colonial zoanthids employ this chemical weapon to deter predators and also during spatial competition. The abundance of this toxin and its potency (LC_50_) in *Artemia salina* were found to vary within regions of single colonies and among reef sites [80]. Within a colony, crude organic extract (COE) was found to be most potent in peripheral regions, where encounters with competing organisms were most likely, compared to central regions. Similarly, differences in COE potency was observed among four Caribbean reef sites; this variability was not significantly associated with differences in reef biodiversity and depth. However, there was a positive correlation between COE yield and reef diversity at one site, providing preliminary evidence for interplay between increased competition and increased demand for toxins [80]. As palytoxin is unlikely to fully account for these observed differences in toxicity, with any number of other chemical components also present in the COE, further research will be required to substantiate these findings and explore the role of environmental factors in driving intracolony and intercolony toxin variability.

Within colonial hydrozoans, functions such as prey capture, defence, digestion and reproduction are divided among three polyp groups—gastrozooids, gonozoids and dactylzooids [81]. Through differential gene expression analysis, genes with key roles in generating functional and structural diversity within colonies have been identified. Furthermore, toxin genes were found to be differentially expressed between specialised polyp types [82]. Using RNA-seq analysis, 75% of putative toxin genes identified were found to be significantly differentially expressed between zooid forms in *Hydractinia symbiolongicarpus*. While toxin families may be present across multiple zooids, the overall venom composition reflected and supported the functions of gastrozooids, gonozoids and dactylzooids in digestion, reproduction and feeding, respectively. Hence, subdivision of labour within colonial hydrozoans is enabled by toxin cocktails that are unique to each zooid type [63,81]. Furthermore, toxin arsenals of functionally distinct structures/tissues are also likely to have diverged in their composition.

## 6. Shared and Specialised Morphology

Cnidarians are characterised by a simple body plan composed of two cellular layers—the epidermis (ectoderm) and gastrodermis (endoderm)—separated by the largely acellular mesoglea [41,83]. Nematocytes, the ‘stinging cells’ that house nematocysts, are distributed throughout the ectoderm (epiderm) and potentially the endoderm [17,84]. Within both the polyp and medusa stages, the epidermis and gastrodermis layers are organised around a central space, termed the gastrovascular cavity [85]. If present, mesenteries are the major anatomical feature of the gastrovascular cavity and are predominantly responsible for digestion [83,85,86]. Within the mesenteries are discrete regions of tissue that serve a reproductive function but are not true gonads [85,86]. Complex internal anatomy and endodermal nematocysts are notably absent in Hydrozoa [87]. Tentacles are present at the open end of this column and are arranged in two or more cycles surrounding a single opening, the mouth, which serves as both point of entry and exit from the gastrovascular cavity [86].

In polyps, the other end can taper to form a bulb-like structure (physa) but more often expands to form the pedal disc [83,85,86]. These structures allow the animal to burrow or attach to a substrate, respectively [83,86]. Staurozoa, Cubozoa, Scyphozoa and Hydrozoa undergo a transformation from tubular polyps into bell-shaped medusae, their adult form. This metamorphosis comprises an upside down orientation, which is accompanied by a number of other adaptations including increased nematocyst diversity [88]. Medusa of Staurozoa are distinguished from other classes in that they lack mobility and instead remain attached via a peduncle [39]. Thus, even when examining basic morphology, significant diversity occurs within Cnidaria.

**Figure 3:**
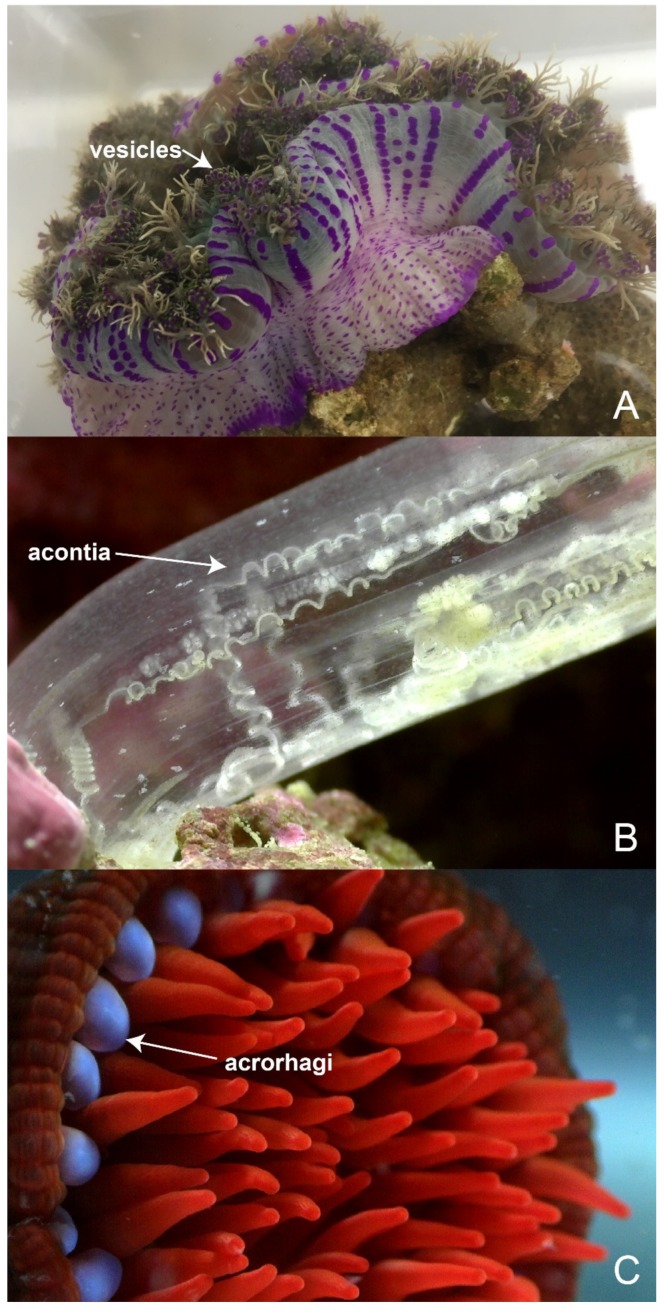
These images show a number of morphological structures used for defence or intraspecific competition by sea anemone species. (**A**) Nematospheres are defensive structures from *Heterodactyla hemprichii*, (**B**) acontia are long thin threads used as defensive structures in *Exaiptasia pallida* and (**C**) acrorhagi are used in territorial fighting in *Actinia tenebrosa.*

Structures with solely defensive or intraspecific aggressive functions are also observed in Actiniaria (class Anthozoa, subclass Hexacorallia). Sea anemones from the genus *Heterodactyla* are characterised by nematospheres (Figure 3A), spherical specialised tentacles associated with an endocoel at the oral disc margin [89]. These nematocyst-dense spheres are presumed to have a defensive role, with analogous structures (vesicles) observed on the column of *Phyllodiscus semoni* [89]. The free edge of mesenterial filaments may also form long thread-like structures (acontia) in a subset of sea anemones (Figure 3B) [90]. Acontiate anemones, including *Telmatactis* spp., eject these structures through the mouth or holes in the column (cinclides) when threatened by predators [91]. Tube anemones also possess acontia [87], although, given their ability to retract within their tube, they may not rely upon these weapons to the same degree.

In addition, a number of structures utilised exclusively for intraspecific aggression are recorded in Actiniaria. Catch tentacles are morphologically and functionally distinct from feeding tentacles, with development induced by proximity to non-clones [92]. If these non-permanent catch tentacles come into contact with another anemone during an aggressive encounter, the holotrich-rich tip remains behind, causing necrosis [93,94]. Sweeper tentacles are the morphological and functional equivalent of catch tentacles found in scleractinian corals and octocorals, and develop in response to competing coral colonies [94,95,96]. Likewise, acrorhagi (Figure 3C), the aggressive organs exclusive to family Actiniidae and located at the tentacle-column margin, cause necrosis in rival anemones [35,36,37,38,94,97]. The diverse functions fulfilled by morphological structures in hexacorallians may underpin similarly diverse venom profiles across different tissues.

## 7. Functional Anatomy and Venom Variation

Previous characterisation of cnidarian venoms has relied largely on tentacle tissue, where the greatest concentration of nematocytes can be observed [17,40,98,99,100]. However, this disregards the widespread nature of nematocytes and provides only a glimpse of the complexity and dynamic nature of the venom landscape. Venom analyses utilising next generation sequencing (NGS) or proteomics offer evidence for the presence of toxins in various morphological structures where nematocytes are found, such as the actinopharynx, mesenterial filaments, column and physa [50,84,101]. Furthermore, Bastos et al. [102] reported that tentacle extracts from *Bunodosoma cangicum* (Anthozoa; Hexacorallia; Actiniaria) were unable to induce apoptosis in zebrafish hepatocytes *in vitro*, but column vesicular extracts from the same organism exerted haemolytic and apoptotic effects, consistent with cytolytic toxins. Likewise, some neurotoxins are localised to ectodermal gland cells rather than nematocytes in the sea anemones *Nematostella vectensis* and *Anthopleura elegantissima* [103]. Therefore, it appears that the regulation of venom composition across tissue types is considerably more complex than the relatively simple structure of these animals would suggest.

**Figure 4:**
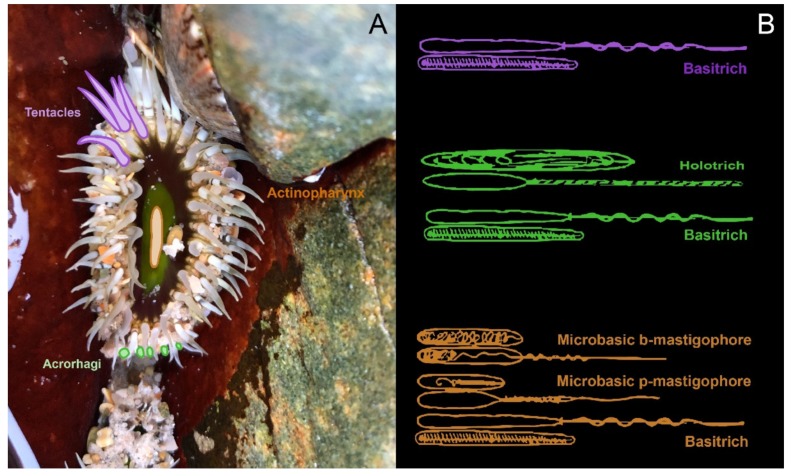
The nematocyst profiles of various tissues in *Oulactis muscosa*, based on cnidom data [105]. (**A**) Tentacles, acrorhagi and actinopharynx of *O. muscosa* are shaded purple, green and orange, respectively; (**B**) the corresponding nematocyst types present in each region are shown in the same colour.

While there can be a shared pool of nematocyst types within a genus, species can be distinguished by variable patterns in the size and localisation of nematocysts [104]. The nematocyst populations of discrete anatomical regions have been detailed in several sea anemone species [90,105,106,107] as well as jellyfish [108,109,110,111], hydromedusae [104], tube anemones [112] and corals [113]. Through these studies, it has become apparent that nematocyst densities vary across the body plan (Figure 4) and that different structures have different proportions of nematocyst types [84,114]. While most tissues contain an assortment of different nematocysts, there are some nematocyst types that are confined to a single tissue, as is the case with very large P-mastigophores and acontial filaments [113]. How these specific nematocysts enhance the predator deterrent power of acontia has yet to be explored, but these observations suggest that the distribution of nematocysts is in part related to the differing functions of tissues.

The relationship between nematocyst and toxin expression profiles provides additional insight into the functional basis of nematocyst variation. Fast-performance liquid chromatography has been used to verify that a difference in nematocyst type is correlated with a difference in venom profiles in *C. fleckeri* [109]. Additionally, homologues of a single protein toxin (the actinoporin equinatoxin) were found to be restricted to a specific nematocyst type in *Hydra magnipapillata* [115]. However, even within a single nematocyst type, subpopulations can be distinguished based on toxin expression profiles [84]. These data, in combination with recent results showing strong differential expression of Cnidarian-specific genes (including those encoding toxins) among different cell types [116], provide evidence for the region-specific production of venoms in a single organism.

The dynamic landscape of sea anemone venoms has been explored in multiple studies. *Nematostella vectensis*, the starlet anemone, is a leading cnidarian model organism owing to the availability of a genome sequence and its ability to be cultured within a laboratory environment [117,118]. Many of the insights into spatiotemporal expression of toxins across the complex life cycle of cnidarians and the development of cnidocytes are based on this species [84,103,116,119]. Recognition of venom arsenal changes with developmental stage and an alternative mechanism of envenomation via ectodermal gland cells are examples of the discoveries made from the study of this species [84,103].

The tissue-specific nature of venom composition in actiniarians has also been explored using four species from the superfamily Actinioidea: *Actinia tenebrosa*, *Anemonia sulcata*, *Heteractis crispa* and *Megalactis griffithsi* [16,50]. Comparison of toxin-like genes across the tentacles, mesenteries and column in *A. sulcata*, *H. crispa* and *M. griffithsi* revealed that the expression of toxins differs among tissues [50]. Toxin expression was consistently lowest in the column and highest in the tentacle or mesenterial filaments depending on the species [50]. This highlights that, while many toxins are expressed throughout the body, tissues with a primary role in envenomation are characterised by an upregulated expression of venom components. Interestingly, toxins from tentacles and mesenterial filaments also show convergence to proteins from other venomous clades, including spiders, snakes, wasps, cephalopods and cone snails [50]. Many of these are among the most highly expressed transcripts within a tissue, such as those with high sequence homology to calglandulin (snake) and venomous translationally-controlled tumour protein (TCTP) homologues (spider and snake), which function in secretion of toxins from the venom gland [120] and the inflammatory activity of venom [121,122], respectively. While these studies emphasise the differences in venom profiles across different tissues in sea anemones, metalloproteases and sea anemone type 2 potassium channel Kunitz-type toxins consistently had the greatest number of transcripts [50], supporting a degree of conservation in toxin expression within superfamily Actinioidea.

Building upon this, it has been demonstrated that toxin expression profiles show different degrees of similarity across tissues in *Actinia tenebrosa*. In this anemone, tentacles and acrorhagi share greater toxin expression similarity with each other than with mesenteries [16]. Furthermore, functional specialisation of venoms in each tissue type is supported by expression of toxin and toxin-like genes and gene ontology (GO) enrichment analysis [16]. Thus, the biological functions of a tissue seem to drive the composition of tissue-specific venom profiles and functionally similar tissues are more likely to have similar toxin expression profiles. However, it cannot be discounted that developmental constraints of the tissue are responsible for, or also contribute to, this expression pattern rather than just the biological function.

## 8. Characterising Toxin Expression Patterns

The diminishing cost and technological advances in sequencing technologies will result in more ‘omics’ datasets for venomous taxa becoming available in coming years, highlighted by the recent publication of multiple cnidarian genomes [118,123,124,125,126,127]. These genomic data represent a rich resource for comparative studies and the elucidation of venom evolution. Functional characterisation is still required for many of the currently identified toxins as this cannot be ascertained from a genome, transcriptome or proteome alone [128,129]. However, by studying toxins in conjunction with their expression patterns, invaluable inferences can be drawn regarding their potential ecological significance.

Platforms for studying toxins *in situ* include *in situ* hybridisation, immunohistochemistry, matrix-assisted laser desorption/ionisation mass spectrometry imaging (MALDI–MSI, henceforth simply MSI) and potentially spatial transcriptomics. Of these, *in situ* hybridisation (ISH) has been used to detect peptide and protein toxins in venomous species since the 1990s [130,131] and remains a leading technique to visualise toxins. The basis of ISH is that the location of a nucleic acid can be visualised using a complementary labelled probe specific to the gene or protein of interest [132]. Elucidating patterns of toxin gene expression across cell types and ontogenetic stages has been achieved through the application of ISH approaches in the model sea anemone species *N. vectensis* [84,101,133]. Furthermore, this approach has enabled the identification of novel and recruited genes with a nematocyte-specific expression pattern [119]. Conversely, immunohistochemistry (IHC) detects the location of peptides in an organism by exploiting antigen–antibody interactions, with the success of IHC dependent upon developing an antibody that is specific and fit for purpose, which is not without its challenges [134]. IHC can be used to complement findings of ISH studies—for example, through a combination of ISH and IHC, it was established that glycerotoxin expression is restricted to a subset of cells in the pharyngeal lobes of bloodworm venom apparatus [135]. Additionally, IHC has been used to demonstrate localisation of sticholysins to tentacles and mesenterial filaments in *Stichodactyla helianthus* [136] and Nv1 (*N. vectensis* toxin 1) to the ectodermal gland cells of *Nematostella vectensis* [103]. However, the probes utilised in both methods are developed for a single target nucleic acid or peptide within a single species, and thus the broad application of these technologies beyond model species is somewhat limited.

In contrast to targeted approaches, high-throughput omics technologies aim to capture the entire DNA, RNA or protein complement within a cell, tissue or organism [137,138]. In addition, MSI offers the opportunity to map the distribution of hundreds to thousands of peptides within a histological tissue section simultaneously, without the need for peptide isolation [139]. Therefore, MSI offers the opportunity to analyse peptide mixtures and evaluate peptide localisation for any species. This approach has been applied to the imaging of venom toxins from sea anemones, snakes and centipedes to date [16,25,140,141,142,143,144]. Identification of venom components directly from MSI spectra, however, remains non-trivial. MSI of toxins is therefore most informative when analysed in light of a venom peptidome obtained by more “traditional” venomic approaches, such as combined transcriptomic and venom proteomic analyses [25]. It has also contributed to understanding the variable tissue expression patterns of toxins within order Actiniaria, having been employed to visualise both widely distributed and highly localised toxins in *A. tenebrosa* [16,25,140]. Taking into account the presence of enzymes within sea anemone venom, the use of MSI as a novel assay to investigate the regulation of enzyme activity also confers considerable utility [144].

## 9. Conclusions

Phylum Cnidaria represents the oldest extant venomous lineage and includes many medically important species, such as the box jellyfish *Chironex fleckeri*. However, the venom of this group remains relatively understudied compared to several of their terrestrial venomous counterparts. It has been established that, in response to environmental stimuli, a single venomous animal can produce multiple venoms with distinct compositions. In cnidarians, these multiple venom profiles are driven in part by pressures related to geographical location as well as ontogenetic stages and associated dietary shifts. However, the colonial organisation of some taxa and the distribution of ‘stinging cells’ throughout the entire body plan augment the complexity of venom production and its regulation.

While venom is used to perform biological functions across every anatomical region in sea anemones (order Actiniaria, subclass Hexacorallia, class Anthozoa), the distinct requirements of each tissue necessitate the expression of unique tissue-specific venom cocktails. In particular, the highly specialised structures exclusively utilised for intraspecific aggression or defence in actiniarians are likely to be accompanied by equally specialised venom profiles. Through recent technological advances which have given rise to methods such as MSI, it is now possible to visualise these spatiotemporal patterns of venom constituents on a large scale. Therefore, by studying toxin distribution in conjunction with knowledge of the functions performed by specific tissues, it is possible to formulate hypotheses on the ecological significance of these peptides without additional functional data, which are often obtained through ecologically non-relevant pharmacological assays. Thus, actiniarians represent a unique opportunity to study toxin pharmacology, structure, and evolution in light of their endogenous functions, due to the discrete ecological roles of the different sea anemone tissues.
